# Effects of capitation payment on utilization and claims expenditure under National Health Insurance Scheme: a cross-sectional study of three regions in Ghana

**DOI:** 10.1186/s13561-018-0203-9

**Published:** 2018-08-27

**Authors:** Francis-Xavier Andoh-Adjei, Bronke Boudewijns, Eric Nsiah-Boateng, Felix Ankomah Asante, Koos van der Velden, Ernst Spaan

**Affiliations:** 1 National Health Insurance Authority, PMB Ministries Post Office, 36-6th Avenue, Ridge, Accra, Ghana; 2 Radboud Institute for Health Sciences, Department for Health Evidence, Radboud University Medical Centre-Netherlands, Nijmegen, Netherlands; 3 Institute of Statistical, Social and Economic Research (ISSER) University of Ghana, Legon, Accra Ghana; 4 Radboud Institute for Health Science, Department for Primary and Community Health, Radboud University Medical Centre-Netherlands, Nijmegen, Netherlands

**Keywords:** Capitation payment, Health service utilization, Claims expenditure, Cost of health care services

## Abstract

**Introduction:**

Ghana introduced capitation payment under National Health Insurance Scheme (NHIS), beginning with pilot in the Ashanti region, in 2012 with a key objective of controlling utilization and related cost. This study sought to analyse utilization and claims expenditure data before and after introduction of capitation payment policy to understand whether the intended objective was achieved.

**Methods:**

The study was cross-sectional, using a non-equivalent pre-test and post-test control group design. We did trend analysis, comparing utilization and claims expenditure data from three administrative regions of Ghana, one being an intervention region and two being control regions, over a 5-year period, 2010–2014. We performed multivariate analysis to determine differences in utilization and claims expenditure between the intervention and control regions, and a difference-in-differences analysis to determine the effect of capitation payment on utilization and claims expenditure in the intervention region.

**Results:**

Findings indicate that growth in outpatient utilization and claims expenditure increased in the pre capitation period in all three regions but slowed in post capitation period in the intervention region. The linear regression analysis showed that there were significant differences in outpatient utilization (*p* = 0.0029) and claims expenditure (*p* = 0.0003) between the intervention and the control regions before implementation of the capitation payment. However, only claims expenditure showed significant difference (*p* = 0.0361) between the intervention and control regions after the introduction of capitation payment. A difference-in-differences analysis, however, showed that capitation payment had a significant negative effect on utilization only, in the Ashanti region (*p* < 0.007). Factors including availability of district hospitals and clinics were significant predictors of outpatient health care utilization.

**Conclusion:**

We conclude that outpatient utilization and related claims expenditure increased in both pre and post capitation periods, but the increase in post capitation period was at slower rate, suggesting that implementation of capitation payment yielded some positive results. Health policy makers in Ghana may, therefore, want to consider capitation a key provider payment method for primary outpatient care in order to control cost in health care delivery.

**Electronic supplementary material:**

The online version of this article (10.1186/s13561-018-0203-9) contains supplementary material, which is available to authorized users.

## Background

The World Health Organisation (WHO) [1] has noted that as many as 11% of the population in some countries incur catastrophic expenditures on health each year while about 5% are pushed into poverty; and only about 5–10% of people living in Sub- Sahara Africa and southern Asia are covered by any type of formal social protection compared with 20–60% in middle income countries. In response to this global challenge, Ghana introduced a national health insurance scheme (NHIS) in 2003 to provide financial risk protection for its population [2]. About 95% of the diseases in Ghana are covered by the health insurance scheme. The Scheme achieved 38% population coverage in 2013 [3] and by end of year 2016, the population coverage stood at 39% of projected national population [NHIA Annual Report, 2016. Unpublished]. The NHIS policy makes provision for several groups of people to be exempted from payment of premium. These include children under 18 years old, elderly above 70 years old, Social Security and National Insurance Trust (SSNIT) pensioners, pregnant women, and those identified as indigent (extreme poor). Sources of funding for the NHIS include the National Health Insurance Levy of 2.5%, a 2.5% of formal sector workers’ contributions to the SSNIT Fund, return on investment, premium paid by non-SSNIT contributors and registration fees. In the years 2009–2011 the NHIS experienced increasing expenditure over income [4], confirming prediction by an actuarial team who cautioned in 2005 that with an anticipated increase in membership enrolment and resultant utilization of health care services under the NHIS, if the funding level remained constant, expenditure would outstrip income by the year 2010 [5], a situation which has been partly attributed to both supply and demand side moral hazards [6]. The National Health Insurance Authority (NHIA) commits to address this challenge through provider payment reforms with the view to containing cost while asuring quality of care. This decision syncs with evidence in literature which indicates that one key motivation for provider payment reforms in countries’ health system is the need for countries to improve efficiency in the application of health care resource in order to contain cost escalation without compromising quality [7–9]. Economic theory, however, points to the fact that changes in provider payment methods elicits responses from health care providers that could negatively affect the quality and quantity of service they provide [10, 11].

Key payment methods applied in countries’ health system include, but not limited to, fee-for-service (FFS), diagnosis-related-groupings (DRG) and capitation payments. FFS payment is used to reimburse health care providers based on item of service they provide and it aligns provider income to quantity of service provided [12]. Associated with supplier-induced demand [13], FFS is described as reward for “volume and intensity rather than value”; and “inflationary and ineffective in containing cost” [14] as it create incentives to increase service provision and pushes the financial risk to the payer. According to Rosen, FFS helps generate valuable health information needed to guide clinical decisions and argues that when providers advise patients in their capacity as agents in care delivery, their influence on patients’ actions may only be described as “physician-initiated” demand and not necessarily supplier-induced demand [15]. FFS is widely used in countries such as Belgium, Germany and Japan [16] as well as in many low and middle-income countries. There are, however, moves by countries to adopt the DRG payment as part of measures to address the negative incentives that FFS payment creates [17]. The DRG, on its part, relates diagnosis and treatment to cost and providers are paid a pre-determined rate for services rendered based on illness episode with the initial motivation to provide framework for monitoring quality of care and utilization [18]. It has strong incentive to induce efficiency and cost-containment but requires quality control and monitoring systems to avert any possible perverse incentives [16]. DRG was introduced by the United States Medicare programme to contain an observed increasing health care expenditure [19] and a number of low and middle-income countries, including Ghana, have adopted various forms of DRG methods to reimburse their health care providers [17]. The advantages of DRG notwithstanding, it offers incentives for providers to give patients a more severe diagnosis to attract a higher reimbursement [20]. Currently, many countries combine it with other forms of payment method including capitation payment. Under capitation payment, providers are paid agreed sum of money per listed patient for a period of time to provide pre-determined services for them, with the expectation that capitation payment will promote efficiency in the use of scarce health care resources by controlling volumes of services provided and related cost. Under capitation payment system, both payers and providers of health care services have the benefit of knowing their budgets in advance. The literature has it that, capitation payment helps to eliminate supplier-induced demand associated with fee-for-service payment [12, 15] but also cautions that unless the capitated rate is risk-adjusted, at least by age and sex, providers may provide less care for perceived risk groups on their list [16, 21].

### Provider payment methods within the NHIS

In 2005, the NHIA adopted FFS payment system to reimburse its credentialed providers but had to introduce a Ghana version of DRG which became known as Ghana-Diagnosis-Related Grouping (G-DRG) system in 2008 with the view to containing observed escalating cost that was being experienced under the FFS payment system. The G-DRG method was implemented nationwide and at all levels of care. It is used to pay for both outpatients (OPD) and inpatients (IPD) services while FFS continues to be used to pay for medicines. Under the G-DRG, related diagnoses are grouped together and the average price of care is determined. The switch from FFS to G-DRG was done without any study by the NHIA to understand the underlying causes of the effects of FFS. Likewise, the G-DRG was introduced without any pilot, neither was it subjected to prospective evaluation to assess it’s likely outcome regarding the cost-containment objective for which it was being introduced. Consequently, the G-DRG was later observed to have rather contributed to cost escalation, more than tripling the cost of claims observed under the FFS. OPD attendance on account of health insurance increased from 597,859 in 2005 to 25,486,081 in 2011. This translates into claims expenditure of GHC7.6 m ($3.53 m) in 2005 and GHC548.7 m ($255.2 m) in 2011. Between 2007 and 2009, the average outpatient claims cost increased by nearly 50% from GHC6.93 (US$2.80) to GHC10.11 (US$4.21), and in 2010, outpatient claims accounted for 70% of total claims received by NHIA. This represented 30% of total costs of claims paid by NHIA. Thus, growth in utilization translates into growth in claims cost and whereas one may acknowledge that the growth in service utilization may be due to the yearly growth in membership and the removal of financial barrier to accessing health care service, it is also not certain whether this phenomenon is a genuine one and that, it is not partly due to moral hazard which is associated with social health insurance schemes world-wide. This concern stems from the observation made over the years during claims processing. It has been noticed with concern that overbilling of medicines, inappropriate application of tariff, duplication of claims, lack of diagnostic evidence to back claims, absence of linkage between treatment and diagnosis and treatment outside the defined benefits package are some of the provider-side moral hazards found with claims submitted by service providers for settlement by the NHIA. Further to that, clinical audit activities also revealed irrational prescription of medicines, inflation of quantities of medicine supplied to subscribers, provision of services above accreditation level and overbilling of medicines as some of the issues found with claims submitted by providers to the NHIA for payment. Between 2010 and 2012 an amount of GHC20.103, 976 ($9,307,396)) was found to have been paid as un-earned claims to providers, partly as a result of the afore-mentioned provider-side moral hazards [3]. The Ministry of Health Joint Assessment Team that conducted a holistic assessment of the Ghana Health Sector Programme of Work, 2012 also noted that since its introduction, the NHIS has led to increase in utilization of OPD services across all the regions. The team, however, raised concern that “with the backdrop of doubling OPD per capita rate, 80% of total outpatients being NHIS-insured members and 34% of the population being active NHIS members, the high proportion of OPD attendance could either be a reflection of frivolous use of services by NHIS members or a reflection of high NHIS membership among those in need of services” and concluded that “a positive answer to these questions poses a financial risk to NHIS, and that these issues should be further analysed and addressed” [22].

Thinking through solutions to address these challenges, the NHIA decided to introduce capitation payment for primary outpatient services as part of its provider payment reforms, beginning with a pilot in the Ashanti region that bore the highest cost burden of claims (28% in 2010). Although capitation payment has been found in literature to encourage efficiency [23]: drive down cost [24, 25], serve as critical source of income for providers [26], promote adherence to guidelines and policies [27] and encourage providers to work better and give health education to patients [28], it is also noted to induce reduction in the quantity and quality of care provided [26], encourage skimming on inputs, “dumping” of high risk patients and negatively affect patient-provider relationship [29]. The NHIA was, however, convinced that notwithstanding any un-intended negative effect following the introduction of capitation payment policy, with a robust monitoring and evaluation system, its implementation could contribute to addressing the cost escalation challenge that is being experienced under the G-DRG and fee-for-service payment methods. A key objective for introducing capitation payment method was to control utilization and contain cost of claims paid by the NHIA. This study was, therefore, undertaken to determine whether capitation payment introduced in the Ashanti region of Ghana contributed in controlling utilization and claims expenditure under the NHIS. On the basis of a hypothesis that capitation payment will result in downward trend or slowed growth in utilization and claims expenditure of the NHIA, we developed the following research question to guide the study: *Does capitation payment have effect on utilization of health care services and related cost at NHIS-credentialed facilities in Ghana?*

## Methods

### Study setting

Three regions, namely Ashanti, Volta and Central, were purposefully selected for the study. Capitation payment policy which is the subject of study was first introduced in the Ashanti region in 2012 and was, therefore, selected as the “intervention” region for the study. Per the NHIA’s implementation plan, the policy was to be implemented in the Volta region in 2016 and in the Central region in 2017. This study being part of a broader monitoring and evaluation system designed to follow the implementation to identify any un-intended effects, we purposively selected the Volta and Central regions that were being prepared for the next implementation phase were selected as “control” regions for this study to enable measurement of effect of the payment policy in Ashanti. Details of characteristics of the three regions are provided in Additional file 1.

### Study design and data

The study was cross-sectional using a non-equivalent pre-test and post-test control group design (before and after study) which according to Creswell [30] is a popular approach to quasi-experiments where both the intervention and control groups are selected without random assignment. Using multiple non-equivalent comparison groups rather than one also increases the ability to explore more threats to the causal inferences [31]. We used aggregated secondary data on utilization and claims expenditure from the NHIA database as validated by the NHIA Actuarial Directorate covering the years 2010–2014. The dataset (Additional file 2) contains district level information on health care utilization for OPD and IPD. Costs are also registered separately for OPD and IPD and are both further split into service and drugs costs. Data on the demographic and socio-economic factors were obtained from records of the Ghana Statistical Service (GSS) [32].

### Statistical data analysis

In total, data from all the 52 districts in three regions were used for analysis: 24 in Ashanti region, 13 in Central region and 15 in Volta region. Descriptive analysis using Microsoft Excel 2007 was performed to examine trends in both OPD and IPD utilization and cost in the intervention region, Ashanti and the two control regions, Central and Volta over a 5-year period, 2010–2014. Utilization and claims expenditure per capita were also analysed by dividing total utilization and total claims expenditure for both OPD and IPD by the respective number of NHIS members in each region. A generalised linear regression model was performed to determine differences in OPD utilization and claims expenditure between the intervention region and the two control regions before and after implementation of the capitation payment system in the Ashanti region. This model was also used to identify predictors of OPD utilization. In addition difference-in-difference analysis (DID) was also conducted using the total district level OPD utilization and cost in all the three regions for each of the study period to estimate the effect of capitation payment system. In both multivariate analyses, the outcome variables were OPD utilization measured in number of visits per year, and OPD costs in Ghanaian Cedi (GHS). Costs were converted from GHS to US dollars (USD) using historical exchange rates to correct for inflation. The explanatory variables were *region*, and assigned a value of 1 if Ashanti, 2 if Central, and 3 if Volta; *time* when the intervention started, a binary variable with a value of 0 for years before 2012 and 1 for years after 2012; *treated*, a binary variable with value of 0 for total OPD health care utilization in the control regions, (Central and Volta) and 1 for total OPD health care utilization in the intervention region (Ashanti); and “*did*”, an interaction between time and treated variables (effect). The dependent variables, OPD health care utilization and OPD cost in the DID models were transformed into logarithmic variables to minimize the problem of skewness. In all the regression models, post-estimation diagnostic checks were also conducted using variance inflation factor (VIF) and co-efficient correlation matrix (Additional file 3) to address the issue of multicollinearity. All covariates that showed VIF estimation of 3 or more were systematically excluded from the models. The analyses were carried out using Excel version 10 and Stata version 13 and a threshold of *p* < 0.05 set for statistical significance.

## Results

### Trends in utilization

Utilization of OPD services at NHIS-credentialed facilities in Ashanti region (intervention) doubled from 3.74 million to 7.49 million visits between 2010 and 2011, the years before the start of implementation of capitation payment but reduced to 4.1 million (− 44.9%) in 2012. Between the years 2013 and 2014 which follow the start date of capitation implementation, utilization increased from 4.29 million to 5.02 million (17.0%) (Table 1). The two control regions, however, exhibited contrasting patterns between them. Utilization in the Central region increased from 1.34 million to 2.42 million visits (80.3%) between 2010 and 2011, and also increased from 1.94 million to 2.54 million visits (30.7%) between 2013 and 2014. In the Volta region, utilization more than doubled from 903,379 to 1.99 million visits between 2010 and 2011 but declined from 2.8 million to 2.7 million visits (0.8%) between 2013 and 2014. Results of the generalized linear regression model showed significant difference in utilization between the intervention region and the control regions, F (2, 49) =6.62, *p* = 0.0029 before implementation of capitation in 2012 (Additional file 4). However, no significant difference in utilization was observed between the intervention and control regions 2 years after the introduction of capitation payment in the Ashanti region, 2013–2014: F (2.49) =0.13. *p* = 0.8755 (Additional file 5).

**Table 1:** Trends in utilization by region and service type, 2010–2014

Region	Year	OPD	% Growth	IPD	% Growth	Total	% Growth
Ashanti	2010	3,738,374	–	132,258	–	3,870,632	–
	2011	7,492,825	100.4	380,886	188.0	7,873,711	103.4
	2012	4,127,229^a^	− 44.9	334,508^a^	− 12.2	4,461,737	−43.3
	2013	4,288,590	3.9	305,893	−8.6	4,594,483	3.0
	2014	5,019,149	17.0	405,719	32.6	5,424,868	18.1
Central	2010	1,344,301	–	49,490	–	1,393,791	–
	2011	2,424,202^a^	80.3	102,484^a^	107.1	2,526,686	81.3
	2012	1,990,644	−17.9	81,071	−20.9	2,071,715	−18.0
	2013	1,940,097	−2.5	131,093	61.7	2,071,190	0.0
	2014	2,535,207	30.7	125,215^a^	− 4.5	2,660,422	28.4
Volta	2010	903,379	–	66,626	–	970,005	–
	2011	1,994,868	120.8	220,441^a^	230.9	2,215,309	128.4
	2012	1,696,435	−15.0	133,838	−39.3	1,830,273	−17.4
	2013	2,754,035	62.3	229,707	71.6	2,983,741	63.0
	2014	2,731,830	−0.8	173,871^a^	− 24.3	2,905,701	−2.6

^**a**^Indicates that for these values inter- or extrapolation was used for one or more of the districts involved

### Trends in costs of utilization

Table 2 showed trends in cost of utilization in all the three regions over the 5-year study period. Claims expenditure in relation to OPD utilization in Ashanti region increased from $49.54 million to $79.42 million (60.3%) before the introduction of the capitation payment policy, dropped by more than half to $38.7 million in 2012 which is the year capitation payment was introduced; and increased again in 2013 from $51.39 million to $63.85 million (24.2%) in 2014, the years after its introduction. In the Central region, claims expenditure related to utilization increased from $15.95 million to $22.93 million (43.7%) between 2010 and 2011 and declined from $21.39 million to $19.53 million (8.7%) between 2013 and 2014. Claims expenditure in the Volta region increased from $11.08 million to $17.05 million (53.9%) between 2010 and 2011, and from 16.31million to 20.52million (25.8%) between 2013 and 2014. The generalized linear regression model showed significant difference in claims expenditure between the intervention region and control regions in the pre capitation period, 2010–2011, F(2, 49) = 9.65, *p* = 0.0003 (Additional file 6) and post capitation period, 2013–2013; F (2, 49) =3.56. *p* = 0.0361 (Additional file 7).

**Table 2:** Trends in utilization cost by region and service type, 2010–2014

Region	Year	OPD cost (USD)				IPD cost (USD)			% Growth
		Service	Drugs	Total	% Growth	Service	Drugs	Total	
Ashanti	2010	23,599,737	25,935,899	49,535,636	–	4,982,751	9,435,406	14,418,157	–
	2011	37,256,361	42,166,756	79,423,117	60.3%	9,307,289	4,162,678	13,469,967	−6.6%
	2012	14,563,585	24,171,523	38,735,108	−51.2%	16,131,540	5,929,841	22,061,381	63.8%
	2013	17,712,047	33,680,387	51,392,434	32.7%	24,467,854^a^	14,967,877^a^	39,435,731	78.8%
	2014	29,917,456	33,932,201	63,849,657	24.2%	16,532,198^a^	8,028,462^a^	24,560,660	−37.7%
Central	2010	6,784,825	9,169,123	15,953,948	–	3,192,779	2,025,999	5,218,778	–
	2011	9,547,133	13,380,507	22,927,640	43.7%	4,422,200	2,047,402	6,469,602	24.0%
	2012	12,570,262	13,473,381	26,043,643	13.6%	5,460,789	2,149,294	7,610,083	17.6%
	2013	11,035,477	10,352,074	21,387,551	−17.9%	5,907,059^a^	2,660,226^a^	8,567,285	12.6%
	2014	9,047,543	10,482,119	19,529,662	−8.7%	4,415,934^a^	1,677,136^a^	6,093,070	−28.9%
Volta	2010	5,249,982	5,829,706	11,079,688		2,492,220	3,551,209	6,043,429	–
	2011	8,658,245	8,396,007	17,054,252	53.9%	3,843,255	2,422,278	6,265,533	3.7%
	2012	9,537,493	8,918,609	18,456,102	8.2%	6,671,738	2,617,453	9,289,191	48.3%
	2013	7,902,767	8,407,392	16,310,159	−11.6%	13,806,537^a^	5,862,988	19,669,525	111.7%
	2014	9,808,899	10,709,757	20,518,656	25.8%	11,159,415^a^	3,164,179^a^	14,323,594	−27.2%

^a^Indicates that for these values inter- or extrapolation was used for one or more of the districts involved

### Effect of capitation payment on utilization and cost

Result of the difference-in-differences analysis showed that capitation payment had a negative effect on both out-patients utilization and related claims expenditure in the intervention region (Tables 3 and 4). The effect was, however, significant on outpatient utilization only (*p* < 0.007). The multivariate regression model revealed that district hospital (*p* = 0.022) and clinic (*p* = 0.027) were significant predictors of health care utilization (Table 5). Other factors such as proportion of the population in poverty, percent of urban population and availability of health centre were also predictors of utilization but their effects were not significant.

**Table 3:** Effect of capitation payment system on OPD utilization in Ashanti region

OPD utilization	Coef.	Robust Std. Err.	t	*p* > t	[95% C.I]		VIF	1/VIF
					Lower	Upper		
time	0.46	0.13	3.58	0.000	0.20	0.71	1.87	0.53
treated	0.6174967	0.15	4.09	0.000	0.32	0.92	2.00	0.50
did	− 0.53	0.19	−2.71	0.007	−0.91	− 0.14	2.87	0.35
_cons	11.45	0.09	116.66	0.000	11.26	11.64		
Mean VIF							2.25	
Number of obs	206							
F(3, 202)	7.72							
Prob>F	0.0001							
R-squared	0.1121							
Root MSE	0.6928							

*VIF* variance inflation factor

**Table 4:** Effect of capitation payment system on OPD cost in Ashanti region

OPD cost	Coef.	Robust Std. Err.	t	*p* > t	[95% C.I]		VIF	1/VIF
					Lower	Upper		
time	0.05	0.14	0.32	0.753	−.024	0.33	1.87	0.53
treated	0.77	0.14	5.34	0.000	0.48	1.05	2.00	0.50
did	−0.19	0.21	−0.92	0.356	−0.62	0.22	2.87	0.35
_cons	13.77	0.09	147.31	0.000	13.57	13.96		
Mean VIF							2.25	
Number of obs	206							
F(3, 202)	13.89							
Prob>F	0.0000							
R-squared	0.1673							
Root MSE	0.76155							

*VIF* variance inflation factor

**Table 5:** Predictors of outpatient utilization of health care services, 2014

Characteristic	Coef.	Std. Err.	t	*p* > t	[95% C.I]		VIF	1/VIF
					Lower	Upper		
Poverty incidence	−0.004	.004	−0.91	0.367	−0.013	0.005	1.37	0.73
Population in poverty	0.000	0.000	1.41	0.166	−0.000	0.000	1.62	0.62
% of urban population	0.004	0.003	1.35	0.183	−0.002	.0108	1.92	0.52
CHPS compound	−0.013	0.013	−0.96	0.340	−0.041	0.015	1.26	0.79
Health centre	0.013	0.016	0.81	0.425	−0.019	0.046	1.38	0.72
Clinic	0.071	0.031	2.29	0.027	0.008	0.134	1.79	0.56
Secondary hospital	0.612	0.257	2.38	0.022	0.093	1.132	1.29	0.78
_cons	11.579	0.230	50.14	0.000	11.113	12.045		
Mean VIF							1.52	
Number of obs	51							
F(7, 43)	7.28							
Prob>F	0.0000							
R-squared	0.5452							
Adj R-squared	0.4680							
Root MSE	0.43532							

*CHPS* Community Health-Based Planning Services, *VIF* variance inflation factor

## Discussion

### Utilization and costs

The main purpose of this study was to assess the effect of capitation payment on the health service utilization and related claims expenditure under Ghana’s National Health Insurance Scheme. A key objective for the introduction of capitation payment was to hold down growth in total health care expenditure by controlling outpatients’ utilization and related expenditure. Our study revealed that outpatient utilization and related claims expenditure increased in both pre and post capitation periods. However, the growth in utilization in the intervention region after the introduction of capitation payment was slower than that of pre-capitation period, suggesting that implementation of capitation payment to control utilization yielded some positive results for the NHIA. This is because in 2012 when the policy was introduced, a negative growth of 44.9% was experienced and thereafter, though there was positive growth of 3.9% in 2013 and 17% in 2014, the trend in growth was slow compared to the period before the introduction of capitation payment. It must, however, be noted that the trend in the control regions were a bit different. While the central region experienced negative growth of 2.5% in 2013, the Volta region experienced a positive growth of 62.3%; and in 2014 Central region recorded a positive growth of 30.7% while the Volta region recorded a negative growth of 0.8% in utilization. With regard to OPD claims expenditure, Ashanti region showed slowed growth of 32.7 and 24.2% in 2013 and 2014, respectively but the control regions exhibited different patterns. Central region experienced negative growth of 17.9 and 8.7% in both 2013 and 2014, respectively but the Volta region experienced negative growth of 11.6% in 2013 and a positive growth of 25.8% in 2014. This phenomenon suggest that factors other than capitation payment policy may have contributed to the trend observed in the Ashanti region. Nonetheless, our findings are consistent with findings in other studies that showed that capitation payment is associated with slower growth of health care expenditures on services that seem profitable under fee-for-service, could contain cost of services and also serve as critical source of income for providers [24–26]. Our findings also corroborate those of a study on capitation payment for primary outpatient services in Zhuhai, China which found a positive effect in controlling costs of health care services [33]. In the China study, the OPD cost increased rapidly over 2 years before the implementation of the capitation payment system but increased at a much lower rate after the implementation, just as our study found that capitation payment had some positive effect in 2012 which was the first year of implementation after which utilization and costs started to increase again but at a slower rate; except that the capitation system in Zhuhai-China was implemented alongside pay-for-performance with a robust monitoring system. This monitoring system which sought to regularly check the behaviours of providers might have contributed to the slowed growth observed in the Chinese study. Similarly in Ghana, clinical audit activities by the NHIA which exposes fraudulent claims by some health care providers and the consequential court action taken against them may have contributed to the observed slowed growth in utilization and claims expenditures. It must, however, be noted that a better effect of the capitation payment could have been achieved but for some design defects of the policy, one being the implementation of the G-DRG alongside the capitation payment at the OPD. The NHIA designed two baskets of services for outpatient care under the capitation payment policy; namely primary outpatients services and “none-primary outpatients services”. With the combination of DRG and capitation payment at the general OPD, it is plausible that potentially primary outpatient services are shifted to the “none-primary outpatients” services and claims made on the latter which cost the NHIA more than the actual cost of the real services rendered. This plausible explanation is based on a study that showed that capitation is associated with the shifting of potentially primary care services to other areas of care [34]. Another possible reason for the increasing outpatient services utilization cost in the post intervention period may be the upward revision of the per capita rate in February 2013 from $0.35 for public facilities, $0.46 for faith-based facilities and $0.65 for private facilities per month to $0.58, $0.79 and $0.84, respectively [4]. During the revision antenatal and post-delivery care were taken out from the basket of services under the capitation payment while payment for medication, which hitherto was part of the capitated fees, was reverted to FFS method. Thus, both ante-natal and post-delivery services were paid by G-DRG method and medicines paid by FFS method, thereby increasing the total cost of OPD services. Other plausible reason for the upward trend in claims expenditure is that providers could be discontent with the new system and try to manipulate their income upwards. This is because studies have found that although health care providers in Ashanti region were aware of the potential advantages and positive attributes of capitation, they perceived the capitation payment and the choice of the region for the pilot to be a politically motivated rather than an intervention aimed at improving efficiency in health care delivery and ensuring quality of care [8, 35]. Consequently, although providers might know the advantages of capitation payment, the negative perceptions they held about it could pose a threat to the policy as observed by Atuoye et al. [36]. The increase in OPD drugs cost for example suggests that providers may have increased their drug prescription to their patients in order to make more money from sale of drugs to the insured. Furthermore, the increase in IPD utilization could be an indication that more patients are referred to a higher level of care under the capitation payment system. In both cases reimbursement for the treatment is by DRG and FFS methods, a situation that suggests that providers receive double reimbursement. Besides the reasons that might have contributed to the OPD utilization and cost in the post capitation period, this study found that availability of district hospitals is a significant contributory factor. This finding is expected because of the general perception that higher level hospitals are better equipped and tend to have more qualified personnel, and therefore, become the preferred choice of majority of insured clients as noted by Andoh-Adjei et al. in a related study [35]. Apart from provider payment method, utilization of health care may also be influenced by demographic, socio-economic and other health system factors [37]. With regard to demographic factors, the literature shows that there is no difference in utilization in Ghana based on gender [38]. A difference was, however, found in utilization for residents in urban areas compared to those in rural areas. For socio-economic factors, there is a difference in utilization of health care service for income groups. Studies [38, 39] have found that in general, the poor benefit less from the NHIS, because they are less often insured but another study also found that the poor who are members of the NHIS utilise services the more [40]. In our study, we noted that factors such as urban population and availability of district hospitals tend to contribute to increasing utilization and cost of outpatient services in the intervention region. One other finding from our study, however, was that higher poverty was correlated with lower OPD service costs, a result that differs from one found in literature [38]. Further studies into this phenomenon will be helpful to policy makers in designing interventions to address the situation.

### Limitations

One major limitation of our study is that individual level data was not available and therefore the use of the aggregate level data led to a smaller sample size and larger standard errors in the regression model. Another limitation is that in the NHIS dataset, some values for utilization and costs were missing but this was only one type and of 1 or 2 years per district. In order to reduce the effect, however, a scatter plot was made for that particular district and type of utilization or costs over the entire time period. A linear regression line with intercept and slope was used to estimate the missing value. Another limitation is that the capitation pilot was started in 2012 and thus, the time period of analysis after capitation is no more than 3 years. This limitation might have contributed to the multicollinearity and inflated standard errors in the models. To see real long term effects, more years of analysis would be needed. These limitations notwithstanding, since the dataset is derived from all the districts in both the intervention and control regions, the results, as presented, provide a reasonably rapid appraisal of the capitation payment policy in the pilot region and, therefore, could serves as prospective evaluation results to guide policy makers on the way forward.

## Conclusion

The study revealed that outpatient utilization and cost increased in both pre and post capitation periods in the intervention region, but the increment in the post capitation period occurred at slower rate, thereby meeting the expectation of the NHIA that capitation would reduce or slow down utilization and related claims expenditures. However, the observed trend in the control regions suggest that factors other than capitation payment policy may have contributed to the slowed growth in utilization and cost in the intervention region. Health policy makers in Ghana may, therefore, want to consider capitation a key provider payment method for primary out-patient care in order to control cost in health care delivery. The current design, however, needs to be reviewed whilst education on the potential benefits of capitation should be intensified among stakeholders and the general public before consideration is given to a nationwide roll-out.

## Additional files

Additional file 1: Characteristics of study settings. (DOCX 17 kb)

Additional file 2: Raw dataset on utilization and claims expenditure. (XLSX 157 kb)

Additional file 3: Correlation matrix of coefficients of regression model for outpatient utilization, 2014. (DOCX 14 kb)

Additional file 4: Linear regression model for outpatient utilization before the intervention. (DOCX 39 kb)

Additional file 5: Linear regression model for outpatient utilization after the intervention. (DOCX 39 kb)

Additional file 6: Linear regression model for outpatient claims cost before the intervention. (DOCX 40 kb)

Additional file 7: Linear regression model for outpatient claims cost after the intervention. (DOCX 39 kb)

## Abbreviations

DID: Difference-in Difference
DRG: Diagnosis-Related Groupings
FFS: Fee-For-Service
G-DRG: Ghana Diagnosis-Related Groupings
GHS: Ghana Cedis
GSS: Ghana Statistical Service
NHIA: National Health Insurance Authority
NHIS: National Health Insurance Scheme
OPD: Out-Patients Department
USD: United States Dollars
WHO: World Health Organisation
