# Supplementary material for: Effects of capitation payment on utilization and claims expenditure under National Health Insurance Scheme: a cross-sectional study of three regions in Ghana
Source: Health Econ Rev. 2018 Aug 27;8:17. doi: 10.1186/s13561-018-0203-9 (PMC6111020; doi:10.1186/s13561-018-0203-9)
Supplement: Supplementary file 1 — Characteristics of study settings. (DOCX 17 kb) [file 13561_2018_203_MOESM1_ESM.docx]

**Additional file 1**

**Study setting: T**hree regions, namely Ashanti, Volta and Central, are purposefully selected for the study.

**Ashanti:** Capitation was first piloted in the Ashanti region of Ghana in 2012. It, therefore, becomes a de-facto intervention region for assessment. It became a candidate region for capitation pilot because it bore the highest claims burden (28%) among all the regions at the time. With a total population of 4,780,380, the region accounts for 19.4 % of the total population of Ghana of which 60.6% is urban. Forty-three per cent (43%) of the population is economically active of which 93.4% is employed. As of 2010, there were 601 NHIS-accredited facilities. The Doctor-population ratio was 1:7,184 while Nurse-population ratio was 1:1,156. Per the GHS 2010 Annual Report, the region recorded 53.4% supervised delivery and 86.5% immunization coverage of PENTA III. It recorded 165 maternal institutional deaths. Institutional maternal mortality ratio (IMMR) was reported as 149 deaths per 100,000 live births. The region had 24 districts offices of the National Health Insurance Authority with 1,585,098 active card-bearing members representing 34% of the regional population in 2010. It recorded out-patient attendance of 5,131,216 in 2010 with health insurance card bearing members accounting for over 80%. Out-patient per capita utilization in the same year was 1.04. With an active membership of 34%, the region accounted for 28% of the total NHIS claims expenditure in 2010.

**Volta:** The Volta region has a population size of 2,118,252, representing 8.6% of national population of which 33.7% is urban. Nearly 3% of the population is economically active of which 96.3% is employed. As of 2010, there were 185 NHIS-accredited providers in the region. Doctor-population ratio was 1:32,605 while Nurse-population ratio was 1:1,090. The region recorded 36.9% supervised delivery and 81.4% immunization coverage of PENTA III. It recorded 82 maternal institutional deaths. Institutional maternal mortality ratio (IMMR) was reported as 209 deaths per 100,000 live births. The region has 15 districts offices of the National Health Insurance Authority and insured membership of 581,305 representing 28% of the regional population in 2010. It recorded out-patient attendance of 1,585,643 with health insurance card bearing members accounting for over 80%. Out-patient per capita utilization in the same year was 0.76. With an active membership of 28%, the region accounted for 6% of the total NHIS claims expenditure in 2010.

**Central:** The Central region has a population of 2,201,863 representing 8.9% of the national population. The urban population constitutes 47.1%. Forty-two per cent (42%) of the population is economically active of which 94.8% is employed. As of 2010, there were 282 NHIS-accredited providers. Doctor-population ratio was 1: 18,218 while Nurse-population ratio was 1:1,200. The region recorded 51.6% supervised delivery and 94.3% immunization coverage of PENTA III. It recorded 82 maternal institutional deaths. Institutional maternal mortality ratio (IMMR) was reported as 149 deaths per 100,000 live births. The region has 13 districts offices of the National Health Insurance Authority and insured membership of 492,715 representing 23% of the regional population in 2010. It recorded out-patient utilization of 1,659,856 with health insurance card bearing members accounting for over 80%. Out-patient per capita utilization in the same year was 0.81. With an active membership of 23% the region accounted for 7% of the total NHIS claims expenditure in 2010.
