# Supplementary material for: Effects of capitation payment on utilization and claims expenditure under National Health Insurance Scheme: a cross-sectional study of three regions in Ghana
Source: Health Econ Rev. 2018 Aug 27;8:17. doi: 10.1186/s13561-018-0203-9 (PMC6111020; doi:10.1186/s13561-018-0203-9)
Supplement: Supplementary file 3 — Correlation matrix of coefficients of regression model for outpatient utilization, 2014. (DOCX 14 kb) [file 13561_2018_203_MOESM3_ESM.docx]

**Additional file 3**: Correlation matrix of coefficients of regression model for outpatient utilization, 2014

| e(V) | Poverty incidence | Population in poverty | % of urban population | CHPS compound | Health centre | Clinic | Secondary hospital | _cons |
| --- | --- | --- | --- | --- | --- | --- | --- | --- |
| Poverty incidence | 1.0000 |  |  |  |  |  |  |  |
| Population in poverty | 0.2376 | 1.0000 |  |  |  |  |  |  |
| % of urban population | 0.1671 | -0.2821 | 1.0000 |  |  |  |  |  |
| CHPS compound | -0.0821 | -0.3360 | 0.0314 | 1.0000 |  |  |  |  |
| Health centre | -0.1219 | -0.4472 | 0.3019 | 0.0802 | 1.0000 |  |  |  |
| Clinic | 0.1438 | -0.1075 | -0.4271 | 0.2500 | 0.0586 | 1.0000 |  |  |
| Secondary hospital | 0.1258 | 0.2377 | -0.1432 | -0.2978 | -0.2483 | -0.2354 | 1.0000 |  |
| _cons | -0.6408 | -0.2216 | -0.5038 | -0.1613 | -0.3511 | -0.0755 | 0.0596 | 1.0000 |
