# Supplementary material for: Effects of capitation payment on utilization and claims expenditure under National Health Insurance Scheme: a cross-sectional study of three regions in Ghana
Source: Health Econ Rev. 2018 Aug 27;8:17. doi: 10.1186/s13561-018-0203-9 (PMC6111020; doi:10.1186/s13561-018-0203-9)
Supplement: Supplementary file 4 — Linear regression model for outpatient utilization before the intervention. (DOCX 39 kb) [file 13561_2018_203_MOESM4_ESM.docx]

**Additional file 4**: Linear regression model for outpatient utilization before the intervention

Ref. Ashanti; 2: Central; 3: Volta
